# Genome-Wide RNA Sequencing of Human Trabecular Meshwork Cells Treated with TGF-β1: Relevance to Pseudoexfoliation Glaucoma

**DOI:** 10.3390/biom12111693

**Published:** 2022-11-15

**Authors:** Anton W. Roodnat, Breedge Callaghan, Chelsey Doyle, Megan Henry, Katarzyna Goljanek-Whysall, David A. Simpson, Carl Sheridan, Sarah D. Atkinson, Colin E. Willoughby

**Affiliations:** 1 Genomic Medicine, Biomedical Sciences Research Institute, Ulster University, Coleraine Campus, Coleraine BT52 1SA, Northern Ireland, UK; 2 Personalised Medicine Centre, Ulster University, Londonderry BT47 6SB, Northern Ireland, UK; 3 School of Medicine, Physiology, National University of Ireland Galway, H91 W5P7 Galway, Ireland; 4 Institute of Life Course and Medical Sciences, University of Liverpool, Liverpool L7 8TX, England, UK; 5 The Wellcome-Wolfson Institute for Experimental Medicine, School of Medicine, Dentistry and Biomedical Sciences, Queen’s University, Belfast BT9 7BL, Northern Ireland, UK

**Keywords:** exfoliation syndrome, exfoliation glaucoma, pseudoexfoliation syndrome, pseudoexfoliation glaucoma, open-angle glaucoma, transforming growth factor beta1, RNA-Seq, transcriptome, trabecular meshwork

## Abstract

Pseudoexfoliation glaucoma (XFG) is an aggressive form of secondary open angle glaucoma, characterised by the production of exfoliation material and is estimated to affect 30 million people worldwide. Activation of the TGF-β pathway by TGF-β1 has been implicated in the pathogenesis of pseudoexfoliation glaucoma. To further investigate the role of TGF-β1 in glaucomatous changes in the trabecular meshwork (TM), we used RNA-Seq to determine TGF-β1 induced changes in the transcriptome of normal human trabecular meshwork (HTM) cells. The main purpose of this study was to perform a hypothesis-independent RNA sequencing analysis to investigate genome-wide alterations in the transcriptome of normal HTMs stimulated with TGF-β1 and investigate possible pathophysiological mechanisms driving XFG. Our results identified multiple differentially expressed genes including several genes known to be present in exfoliation material. Significantly altered pathways, biological processes and molecular functions included extracellular matrix remodelling, Hippo and Wnt pathways, the unfolded protein response, oxidative stress, and the antioxidant system. This cellular model of pseudoexfoliation glaucoma can provide insight into disease pathogenesis and support the development of novel therapeutic interventions.

## 1. Introduction

Pseudoexfoliation syndrome (XFS; MIM: #177650) is a systemic condition characterised by the production of fibrillar exfoliation material (XFM) which can result in several clinical and ocular manifestations including cataract and an aggressive form of secondary open angle glaucoma called pseudoexfoliation glaucoma (XFG) [1]. The prevalence of XFS is estimated to be 60 million people worldwide, of which half will eventually develop XFG [1]. The pathogenesis of XFS and progression to XFG have not been fully elucidated [1,2,3]. Pathognomonic XFM deposits are deposited on several structures in the anterior segment of the eye including the lens, ciliary body, iris and trabecular meshwork in XFG. This XFM consists of elastin, fibrillin, fibulins, latent TGF-β binding proteins, lysyl oxidase like 1 (LOXL1) and other components [4,5]. In XFG, the intraocular pressure (IOP) is elevated, presumably in part due to obstruction of the outflow facility by XFM [6] and changes in the outflow facility consisting of the trabecular meshwork (TM) and Schlemm’s canal (SC). 

XFS and XFG are highly associated with single nucleotide polymorphisms of lysyl oxidase like 1 (LOXL1) [7], a gene which is involved in crosslinking collagen and elastin, and which is also a component of the XFM [5]. Certain risk-alleles in LOXL1 that are highly associated with XFS/XFG are also common in unaffected individuals indicating that other risk-factors play a role in the development of XFS/XFG, such as, older age, exposure to UV radiation [8], reactive oxygen species [9], homocysteinaemia [10] and folate intake [11], dysregulated retinoic acid signalling [12], latitude probably related to UV exposure [8], impaired autophagy [13] and elevation of several signal molecules such as transforming growth factor beta 1 (TGF-β1) in the aqueous humour (AH) [14]. In XFG, TGF-β1 is upregulated both in active form in the AH and in its latent form as part of the large latent complex which binds to the extracellular matrix (ECM) [14]. TGF-β1 is a potent signal molecule associated with fibrosis which can activate several pathways that control cellular processes such as growth, apoptosis, differentiation, cell migration, oxidative stress and extracellular matrix remodelling in a cell-type and cell-context dependent manner [15].

Despite advances in genetic basis of XFS/XFG [2] there is a need for the integration of multiomics data and further experimental work to fully elucidate the molecular mechanisms driving XFS/XFG [2,4,16]. Obtaining disease relevant tissue from human subjects with XFG is challenging [17,18] and researchers have used lens capsule and Tenon’s ocular fibroblasts as a surrogate for the trabecular meshwork [4,14,19]. However, molecular and cellular alterations in the trabecular meshwork drive the reduction in outflow facility and raised IOP seen in XFG. Difficulties in obtaining trabecular meshwork tissue has required the use of postmortem studies [17,18]. Animal models of XFS/XFG are also difficult as pathogenesis is multifactorial and transgenic models are monogenic using Loxl1^−/−^, Loxl1^+/−^, and Loxl1^+/+^ mice, and elevated IOP results from alterations in episcleral venous pressure rather than alterations in the trabecular meshwork. Therefore, stimulating normal human trabecular meshwork cells (HTMs) with TGF-β1 has been proposed as an in vitro model of XFG to determine the molecular mechanisms in a cellular model [20]. While altered TGF-β1 is only one component of the complex cascade driving the pathogenesis of XFS/XFG understanding its role in the trabecular meshwork will provide insight into potential disease mechanisms and therapeutic opportunities.

There is significant overlap between biological processes known to be activated by TGF-β1 [21] and pathological fibrotic changes in XFG such as XFM production [5,14], oxidative stress [6,22], unfolded protein response [23], dysregulated retinoic acid signalling [12] and fibrotic ECM remodelling [20] suggesting that TGF-β1 plays an important role in the pathogenesis of XFG [20]. Therefore, the main purpose of this study was to perform a hypothesis-independent RNA sequencing analysis to investigate genome-wide alterations in the transcriptome of normal HTMs stimulated with TGF-β1 and investigate possible pathophysiological mechanisms driving XFG.

## 2. Materials and Methods

### 2.1. Human Trabecular Meshwork (HTM) Culture and Characterisation

Primary normal human trabecular meshwork (HTM) cells were harvested from donor eyes (*n* = 4) obtained from the Liverpool Research Eye Bank and approved by the local ethics review board (RETH000833). Samples were handled in accordance with the Declaration of Helsinki. Eyes were obtained from the Royal Liverpool University Hospital Mortuary and medical history was unknown; although no donor had previous ocular surgery or a known diagnosis of glaucoma. Donor information is listed in Supplemental Figure S1. Donor eyes were excluded if the maximum post-mortem time exceeded 48 h and HTM cells were isolated using the blunt dissection method as reported previously [24]. Cells were maintained in Dulbecco’s Modified Eagle Media (DMEM)-low glucose (Sigma, Gillingham, UK) supplemented with 10% fetal calf serum (Biosera, Heathfield, UK), 2 mM L-glutamine (Sigma, Gillingham, UK), Pen/Step (Sigma, Gillingham, UK), and 2.5 µg/mL Fungizone (amphotericin B, Sigma, Gillingham, UK). Samples were incubated at 37 °C (5% CO_2_ and 95% humidity). HTM characterisation was carried out as previously described [24] and included upregulated myocilin protein expression in response to dexamethasone treatment (polyclonal rabbit anti-myocilin primary antibody was a kind gift from Dr. W. Daniel Stamer) as previously described by our group [25].

### 2.2. TGF-β1 Treatment and RNA Extraction

Human TM cells between passages 4 and 6 were grown to 80% confluency and growth arrested using serum free medium prior to TGF-β1 stimulation. Cells were stimulated with recombinant human TGF-β1 (R&D Systems, Abingdon, UK) at a concentration of 5 ng/mL for 24 h. Total RNA was extracted from cells using the Qiagen AllPrep DNA/RNA/miRNA Universal Kit (Qiagen, Manchester, UK) following manufacturer’s instructions. The RNA concentration was measured using the NanoDrop 2000 (Thermofisher Scientific, Horsham, UK) and RNA quality was determined by the Bioanalyser 2100 (Agilent Technologies, Stockport, UK) using an RNA 6000 Nano Kit (Agilent, Santa Clara, CA, USA).

### 2.3. Genome-Wide Expression Profiling with RNA Sequencing

RNA-Seq analysis was performed by Novogene (Novogene, Milton, UK) with sequencing libraries generated using NEBNext Ultra TM RNA library Prep Kit (NEB, Ipswich, MA, USA) and mRNA was purified using poly-T oligo-attached magnetic beads. Sequencing was performed on an Illumina platform resulting in a set of FASTQ files containing paired end reads.

### 2.4. RNA-Seq Data Analysis

The resulting reads were subjected to quality control using FastQC v0.11.9 [26]. If needed, adaptor and quality trimming of reads was performed using Cutadapt 3.0 [27] resulting in high-quality trimmed reads with a Phred quality score larger than 30 (corresponding to 0.1% base calling error rate). High-quality trimmed paired reads were aligned to the genome sequence of assembly GRCh38 using the STAR aligner 2.7.6a [28] together with the comprehensive gene annotation for the primary assembly [29]. Quantification of reads per feature (gene) was performed using FeatureCounts 2.0.1 [30] resulting in a table of counts per sample and per gene. Genes with an average read count lower than 50 were filtered out. Using this filtered table, differential gene expression was determined using DESeq2 version 3.15 [31] in R resulting in a gene list containing *p*-value, false discovery rate (FDR) and counts per million reads mapped (CPM) in which CPM values were determined using function fpm in DESeq2. Genes with an FDR < 0.05 and absolute |log_2_FC| > 0.26 (corresponding to at least 20% fold change) were considered to be differentially expressed genes (DEGs) and subsequently used for further analysis.

### 2.5. Functional and Pathway Enrichment Analysis

Using the gene list containing DEGs, an over-representation analysis was performed using Enrichr [32] which uses a modified Fisher exact test to test for over-representation of an input gene list in pathway databases and gene ontologies, beyond what would be expected by chance. Enrichment was determined for KEGG and reactome pathways and for Gene Ontology (GO) molecular functions and biological processes. Pathways or GO terms were assumed to be enriched if both false discovery rate FDR < 0.1 and nominal *p*-value *p* < 0.01. To visualise and cluster the enrichment results, Cytoscape (version 3.9.1) app EnrichmentMap 3.3.4 [33] was used together with AutoAnnotate 1.3.5 [34]. To further investigate and visualise certain interesting enriched KEGG pathways, these pathways were annotated with gene expression results using Pathview 1.30.1 in R [35]. To determine enrichment of relevant gene lists not found in common databases, such as exfoliation material (XFM) genes and XFS GWAS results, custom lists were created and gene expression for these gene lists was determined. For XFM genes, the list published in Supplementary Table S3 of [4] was used. A gene list of XFS GWAS genes was compiled from [36].

### 2.6. LOXL1 Genotyping

A set of LOXL1 exon mRNA sequences encompassing single nucleotide polymorphisms (SNPs) known to be associated with XFG was obtained from dbSNP [37]. To minimise the risk of false positives all sequences were selected to be at least 25 nucleotides long but still short enough to fit well within the length of a read (150 bp). The LOXL1 genotype of each donor with respect to the selected SNPs was assessed by determining the frequency of each detection sequence in all fastq files for that donor. Fastq file processing was done using a bash script followed by R postprocessing to create a heatmap of SNPs per donor.

### 2.7. RNA-Seq Data Validation

Validation of RNA samples was performed on three of the same TM donor cells used in the RNA-seq. Reverse transcription was performed using the Roche Transcriptor First Strand cDNA Synthesis kit (Roche Applied Science, Burgess Hill, UK) using 500 ng of total RNA and anchored-oligo (dt) primers. Using Primer Design Ltd. primer assays (Primerdesign Ltd., Chandler’s Ford, UK), RT-qPCR was performed for the genes: TSPAN2, ADAM19, IER3, CCN2, ADAM12, NOX4, THBS1, LOXL1 and the housekeeping gene GAPDH. RT-qPCR was performed using custom primers (Primer Design Ltd. UK) for target genes (Supplemental Table S1). RT-qPCR was performed on a LightCycler^®^480 real-time PCR system (Roche Diagnostics, Rotkreuz, Switzerland) using the LightCycler^®^ 480 SYBR Green I Master kit according to manufacturer’s specifications (Roche Applied Science,Burgess Hill, UK). All mRNA was measured at CT threshold levels and normalised with the average CT values of a reference gene; GAPDH. Values were expressed as fold increase over the corresponding values for control by the 2^−ΔΔCT^ method. Per sample, two independent experiments were performed, and the average (±SD) results were calculated using GraphPad software (GraphPad Software, San Diego, CA, USA). Data were expressed as the mean values ± SD and graphed using log scale. Statistical significance was analysed using a paired Student *t*-test. Differences in the mean were considered statistically significant if *p* < 0.05.

## 3. Results

### 3.1. Descriptive Features of RNA-Seq Data

On average, sequencing of 8 samples from 4 donors resulted in 46 M 150 bp reads per sample so 23 M paired-end reads in total. The Phred quality score was larger than 30 (corresponding to 0.1% base calling error rate) for most reads but some reads required modest quality trimming using Cutadapt to arrive at quality scores larger than 30. Paired trimmed high-quality reads were mapped to the genome (GRCh38) with an average mapping percentage of 85%. 

### 3.2. LOXL1 Genotyping

Because XFG has strong genetic associations with certain LOXL1 SNPs, all samples were genotyped for three relevant exonic alleles in LOXL1: rs3825942[G > A] coding for LOXL1 p.Gly153Asp, rs1048661[G > T] coding for LOXL1 p.Arg141Leu, and rare protective variant rs201011613[A > T] coding for p.Tyr407Phe (see Figure S1). Other possible but less relevant genotypes for these SNPs were also analysed. This analysis was performed to assess if one of the donors may be at higher risk of developing XFG which may affect responsiveness to TGF-β1 treatment or gene expression in the untreated control because there may already have been premorbid exposure to higher TGF-β1 levels in such a donor. Donors 1 and 4 were heterozygous for allele rs3825942:[G > A] while donor 2 was heterozygous for rs1048661:[G > T; p.Arg141Leu] (see Figure S1). None of the donors was homozygous for a high-risk allele.

### 3.3. Differential Gene Expression in Normal HTM Cells following TGF-β1 Treatment

**Figure 1:**
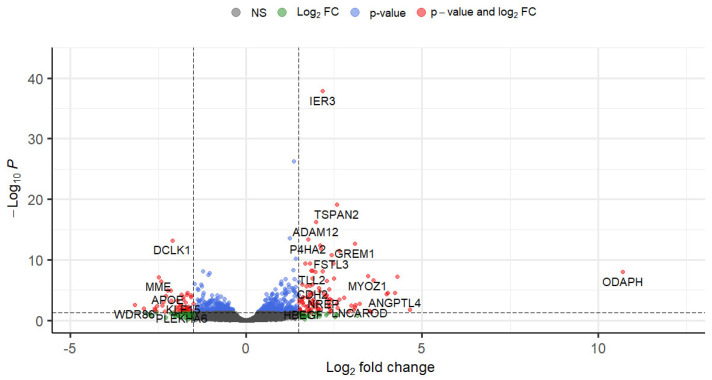
Volcano plot identifying differentially expressed genes in response to TGF-β1. Where grey and green dots = not significant when using false discovery rate corrected for multiple testing (where FDR > 0.05), blue dots represent genes with FDR < 0.05 and |log_2_FC| ≤ 1.5, red dots represent genes that are both significantly expressed and have a high fold-change: FDR < 0.05 and |log_2_FC| > 1.5. There were 143 genes with FDR < 0.05 and |log_2_FC| > 1.5 (marked in red) of which 95 genes were upregulated and 48 genes were downregulated. In this work all genes with FDR < 0.05 and |log_2_FC| > 0.26 have been taken into account (all red genes and most blue genes) which amounts to 689 genes of which 392 genes were upregulated and 297 genes were downregulated.

Reads were aligned to the genome sequence of primary assembly GRCh38 using the primary comprehensive gene annotation. Features with a low number of counts were filtered out resulting in a total of 12,882 aligned genes. Using R package Deseq2, the log_2_FC, log_2_CPM, *p*-value and false discovery rate (FDR) per gene were determined. Figure 1 shows a volcano-plot of all genes with thresholds set at FDR = 0.05 and |log_2_FC| = 1.5.

Of the total set of 12,882 genes, 2179 were significantly differentially expressed based on a nominal per-gene *p*-value (*p* < 0.05) and 689 genes were significantly differentially expressed when correcting for multiple testing (FDR < 0.05) and for fold change by only selecting genes with an absolute fold change of |log_2_FC| > 0.26. Of these 689 genes, 392 genes were upregulated, and 297 genes were downregulated.

**Figure 2:**
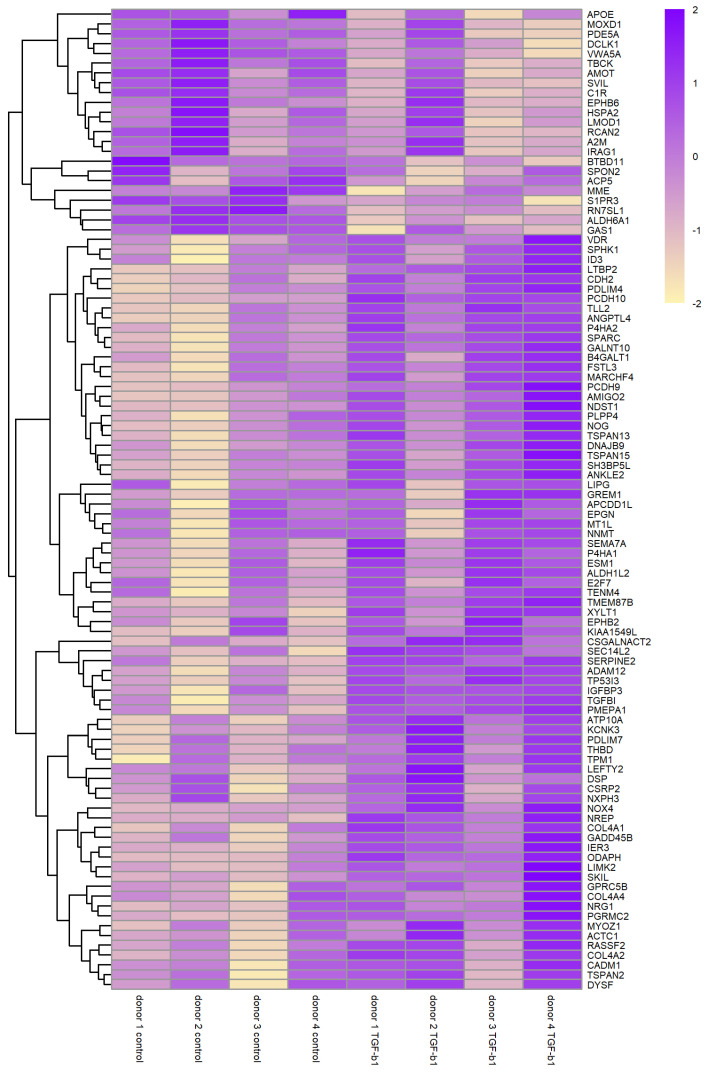
Heatmap showing the expression of the top 100 differentially expressed genes. Expression in log_2_cpm of the top 100 differentially expressed genes ranked by lowest FDR (false discovery rate). The expression pattern of each gene has been scaled to enable comparison between genes and hierarchical clustering of genes has been applied to group genes by normalised gene expression pattern. This heatmap shows correct clustering of upregulated and downregulated genes but also shows biological variability between the donors.

Table 1 shows the top 50 differentially expressed genes ranked by FDR and Figure 2 shows a heatmap of the normalised expression of top 100 genes with the lowest FDR for all four sample-pairs. This heatmap shows correct clustering for upregulated and downregulated genes but it also shows biological variability between the donors, especially donor 2. When column clustering by sample is applied then TGF-β1 treated samples will cluster for donors 1,3,4 but treated donor 2 will cluster together with untreated donor 2 (not shown). A complete table containing all 12,882 genes that were expressed can be found in Supplemental Table S2.

### 3.4. Functional Enrichment and Pathway Analysis Reveals 53 Enriched Functional Clusters

Using the enrichment tool Enrichr, overexpression analysis was performed on the list of 689 significantly differentially expressed genes to identify significantly altered pathways in KEGG and Reactome and enriched biological processes (BP) and molecular functions (MF) in Gene Ontology (GO). Enrichment results were assumed to be significant when *p* value < 0.01 and FDR < 0.1. This analysis resulted in a total of 219 enrichment terms containing the majority of DEGs. Out of a total of 689 DEGs, 261 were not associated with any enrichment term. Several of these genes are long noncoding RNAs such as LINC00565, LINC00906 of which the function may not currently be clear. Additionally, several high ranking genes (when ranked by FDR) do not show up in any enrichment term, for example IER3, TSPAN2, PCDH10, ATP10A, PLPP4, AMIGO2, RN7SL1, TSPAN13, SVIL, PCDH9, MT1L. Table 2 shows the top 40 enrichment results ranked by *p*-value and overall FDR. This subset of all enriched terms mainly points to remodelling of ECM, canonical TGFβ pathway activation via SMADs, the unfolded protein response and regulation of cell proliferation. 

**Figure 3:**
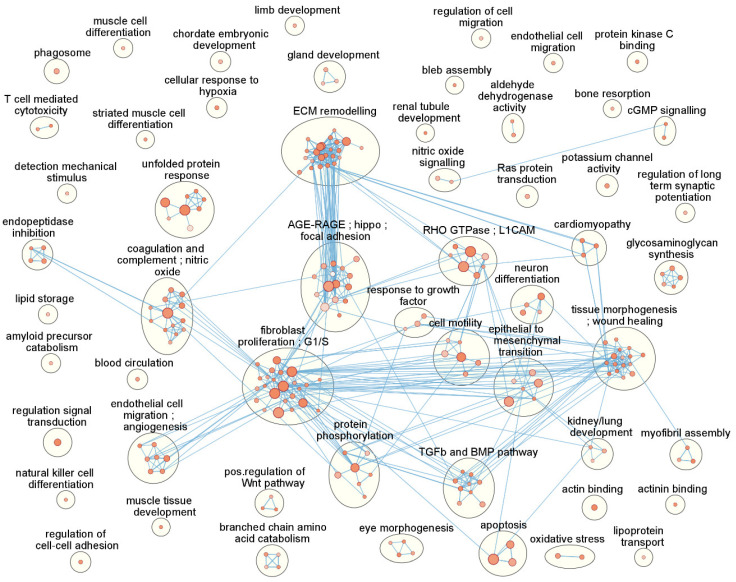
Clustered enrichment results. Clustered enrichment terms based on over-representation analysis (ORA) of a gene list of significantly differentially expressed genes (FDR < 0.05) in normal human trabecular meshwork cells after TGF-β1 treatment. Each node corresponds to an enrichment term. The node intensity represents the nominal *p*-value in a range 0.00 (darkest) to 0.01 (lightest) while the node size corresponds to the number of genes associated with a particular enrichment term. Blue lines represent gene list overlap between enrichment terms above a threshold of 0.25. Clustered enrichment terms have been encircled and annotated.

Using Cytoscape and Cytoscape apps EnrichmentMap and AutoAnnotate, clustering was applied to the set of 219 enriched terms resulting in 55 clusters as shown in Table 3 and graphically in Figure 3. Clustering may provide a better overview of what is actually happening in the cells because similar terms (based on gene overlap) become grouped into one overlapping theme. The largest clusters contain enrichment terms associated with fibroblast proliferation, ECM remodelling, tissue morphogenesis and wound healing, AGE-RAGE, hippo and focal adhesion pathways and terms associated with G1/S cell cycle arrest. All clusters, the associated enrichment terms and their genes can be found in Supplemental Table S3.

As can be observed in Table 2 and Table 3, treatment of normal human trabecular meshwork (HTM) cells with TGF-β1 affects many cellular pathways and biological processes. This study focussed on a subset of enriched clusters, namely the effect of TGF-β1 on exfoliation material, extracellular matrix remodelling, extracellular signal molecules, oxidative stress and modulation of the antioxidant system, the unfolded protein response and retinoic acid signalling because of their relevance to the pathogenesis of XFG.

### 3.5. TGF-β1 Causes Differential Expression of Several Exfoliation Material Components

**Figure 4:**
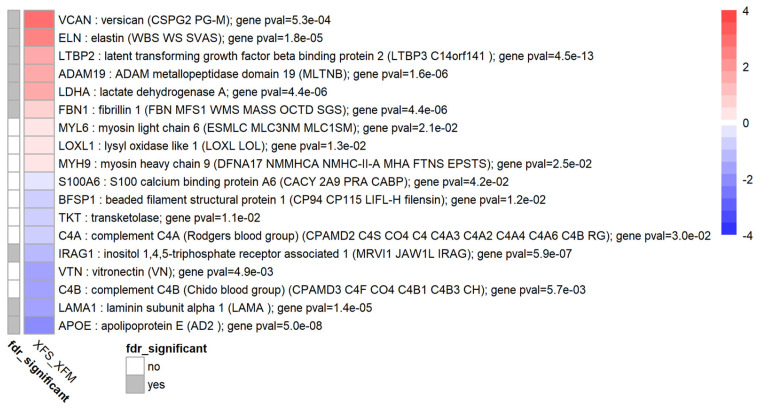
Differential expression of exfoliation material (XFM) genes. Heatmap showing the genes with per-gene *p*-value, where *p* < 0.05, for XFM genes. Heatmap colours correspond to fold change expressed as log_2_FC which has been limited to a range of [−4, 4] for clarity. The grey annotation bar on the left shows if the genome-wide false discovery rate has an FDR < 0.05 (in which FDR is the Benjamini-Hochberg corrected *p*-value) for a particular gene. A total of 9 XFM genes were upregulated and 9 XFM genes were downregulated in response to TGF-β1.

Our analysis showed that several XFM components were upregulated in response to TGF-β1 in normal HTM tissue, see Figure 4. A recently published summary of genes known to be expressed in XFM was used to create a gene list [4]. Genes that appeared to be upregulated with high significance (FDR < 0.05) were VCAN, ELN, LTBP2, ADAM19, LDHA, FBN1, while MYL6, MYH9 and LOXL1 were also upregulated based on their *p*-value. Downregulated genes were IRAG1, LAMA1 and APOE (FDR < 0.05) and S100A6, BFSP1, TKT, C4A, VTN, C4B based on their nominal *p*-value. Clusterin (CLU), another XFM component frequently mentioned in the literature, was not differentially expressed in our dataset.

### 3.6. TGF-β1 Strongly Affects Remodelling of the Extracellular Matrix

**Figure 5:**
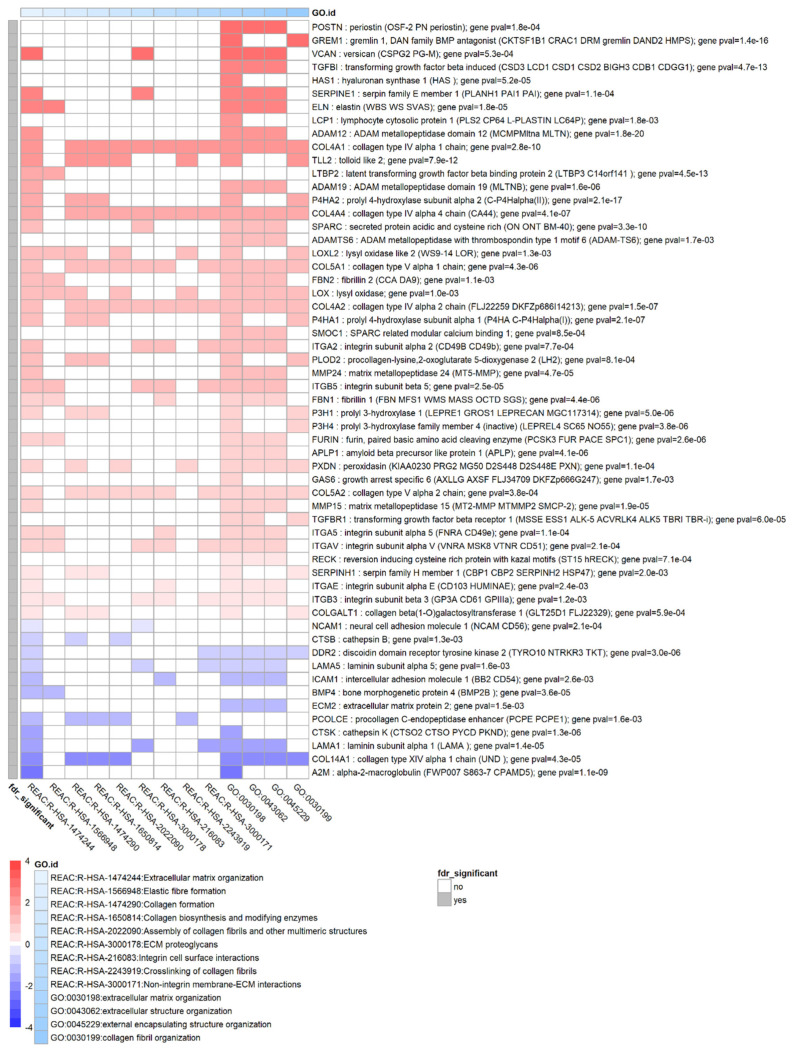
Genes and enrichment terms associated with the cluster ‘ECM remodelling’. Heatmap showing the genes with false discovery rate FDR < 0.05 for genes associated with extracellular matrix remodelling enrichment terms. Heatmap colours correspond to fold change expressed as log_2_FC which has been limited to a range of [−4, 4] for clarity. The grey annotation bar on the left shows if the false discovery rate FDR < 0.05 (in which FDR is the BH corrected *p*-value) for a particular gene which is the case for all genes considered here. To enable visualisation of this cluster, a subset of all enrichment terms in this cluster is shown.

TGF-β1 treatment of HTM resulted in differential expression of many genes associated with the extra-cellular matrix (ECM), either as a component of the ECM or involved in controlling the process of ECM remodelling. Figure 5 shows a heatmap containing a subset of enriched terms in the extracellular matrix remodelling cluster and the differentially expressed genes associated with these terms. Of the collagen family, COL4A1, COL4A2, COL4A4, COL5A1, COL5A2 were upregulated (FDR < 0.05) while COL14A1 was downregulated. Other upregulated ECM components were elastin (ELN) and several fibrillins (FBN1, FBN2). Several members of the integrin family were upregulated: ITGA2, ITGB5, ITGA5, ITGAV, ITGAE, ITGB3. Additionally, several ECM-related enzymes appeared to be differentially expressed due to TGF-β1 such as lysyl oxidases LOX, LOXL2 and metallopeptidases ADAM12 and ADAM19. As previously mentioned, LOXL1 was differentially expressed when considering nominal *p*-value but not when correcting for multiple testing (*p* = 1.3 × 10^−2^). Some key genes with a regulatory role in the TGFβ pathway and in ECM remodelling were also upregulated, namely GREM1 and SPARC. Several genes coding for extracellular matrix proteins known to be involved in TGFβ signalling were also differentially expressed such as latent transforming growth factor beta binding protein 2 (LTBP2).

### 3.7. TGF-β1 Modulates Expression of Extracellular Signal Molecules in HTM Cells

**Figure 6:**
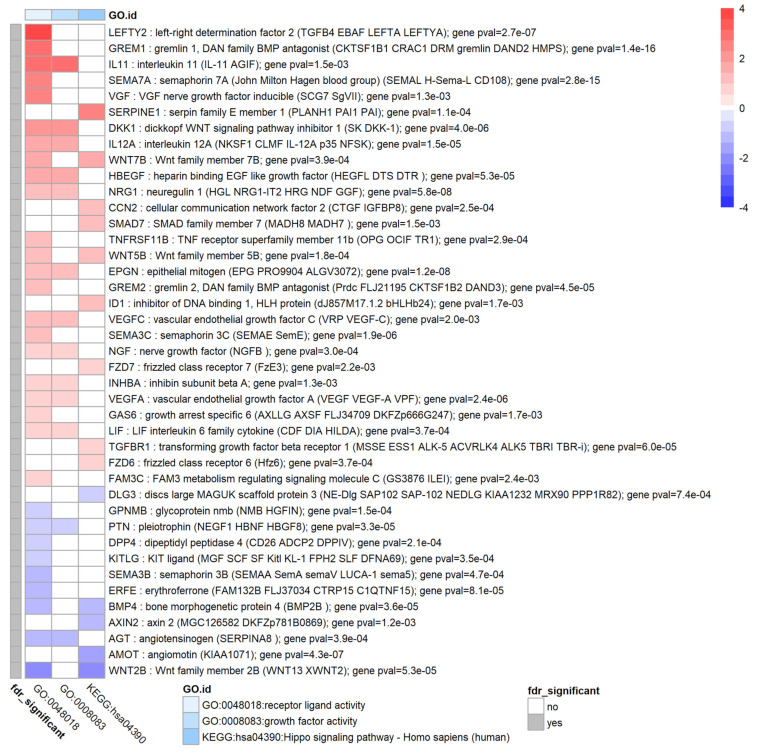
Genes and enrichment terms associated with extracellular signal molecules. Heatmap showing the genes with false discovery rate FDR < 0.05 for genes associated with growth factor activity and hippo signalling pathway. Heatmap colours correspond to fold change expressed as log_2_FC which has been limited to a range of [−4, 4] for clarity. The grey annotation bar on the left shows if the genome-wide false discovery rate is significant, FDR < 0.05 (in which FDR is the BH corrected *p*-value), for a particular gene which is the case for all genes considered here.

Selected enriched terms and their corresponding genes that were found to be associated with extracellular signal molecules such as growth factor and cytokine activity are shown in Figure 6. Several interleukins were upregulated such as IL11, and IL12A. Several growth factors and receptors that are known to be associated with the canonical TGFβ and BMP pathways were also differentially expressed such as LEFTY2, GREM1, GREM2, TGFBR1 (all up) and BMP4 (down). Another key upregulated growth factor was CCN2 (also known as connective tissue growth factor: CTGF). Furthermore, components that are associated with the Wnt pathway were upregulated namely DKK1, WNT5B and WNT2B. TGF-β1 expression itself was upregulated (log_2_FC = 0.6, *p* = 0.04). Additionally, noteworthy is the upregulation of vascular endothelial growth factors VEGFA and VEGFC and furthermore NRG1, ID1, NGF, GAS6, HBEGF and VGF (all upregulated) and angiotensin (AGT, downregulated). Although not showing up in this matrix, other related genes that showed an upregulation were follistatin like 3 (FSTL3, log_2_FC = 2.4, FDR = 1.2 × 10^−8^) and endothelin 1 (EDN1, log_2_FC = 3.2, *p* = 0.02). The latter gene showed a large fold change for some donors but also showed large biological variability between donors.

### 3.8. TGF-β1 Modulates Oxidative Stress and the Antioxidant System in HTM Cells

**Figure 7:**
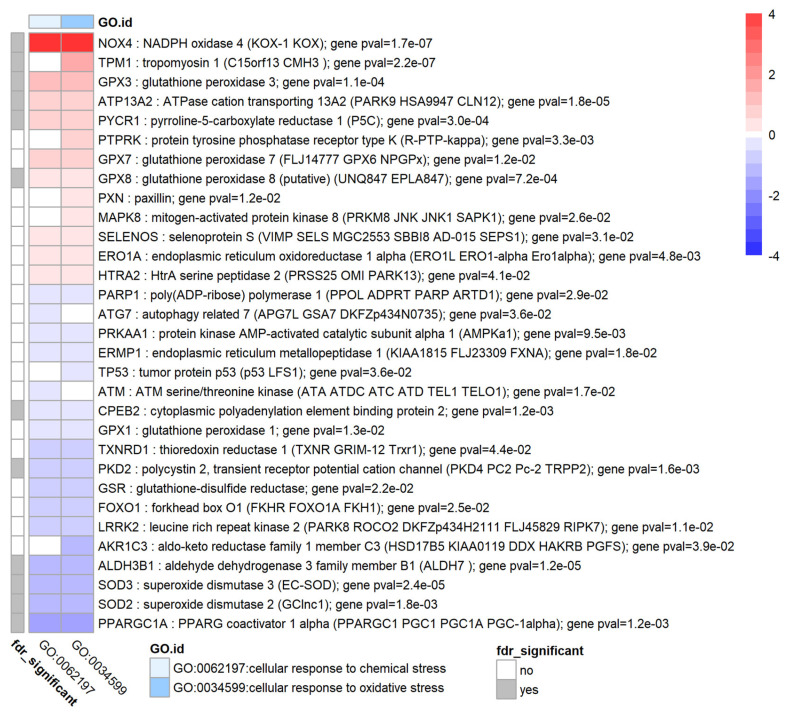
Genes and enrichment terms associated with the cluster ‘oxidative stress’. Heatmap showing the genes with false discovery rate FDR < 0.05 for genes associated with enrichment terms related to oxidative stress. Heatmap colours correspond to fold change expressed as log_2_FC which has been limited to a range of [−4, 4] for clarity. The grey annotation bar on the left shows if the genome-wide false discovery rate has an FDR < 0.05 (in which FDR is the BH corrected *p*-value) for a particular gene.

Figure 7 shows enriched terms and genes associated with cluster ‘oxidative stress’. TGF-β1 treatment of HTM cells clearly induced changes in expression of several genes that are part of the antioxidant system such as glutathione peroxidases (GPX3, GPX7, GPX8: upregulated and GPX1: downregulated), superoxide dismutases (SOD2 and SOD3: downregulated) and glutathione disulfide reductase GSR (downregulated). TGF-β1 also increased oxidative stress and modulated redox signalling by highly upregulating NADPH oxidase 4 (NOX4). Other interesting genes in this cluster were TPM1, ATP13A2, PYCR1 (upregulated) and CPEB2, PKD2, ALDH3B1, PPARGC1A and TP53 (downregulated). To further investigate oxidative stress, the set of differentially expressed genes associated with Gene Ontology term GO:0016209 (antioxidant activity) was determined. This analysis showed that also PTGS2 (COX2) and peroxidasin (PXDN) were upregulated while PTGES and APOE were downregulated. Although not present in the enrichment results shown here, it appeared that several glutathione S-transferase components, namely GSTM4, GSTM3, GSTA4 were also downregulated (*p* < 0.02).

### 3.9. TGF-β1 Causes Upregulation of the Unfolded Protein Response in HTM Cells

**Figure 8:**
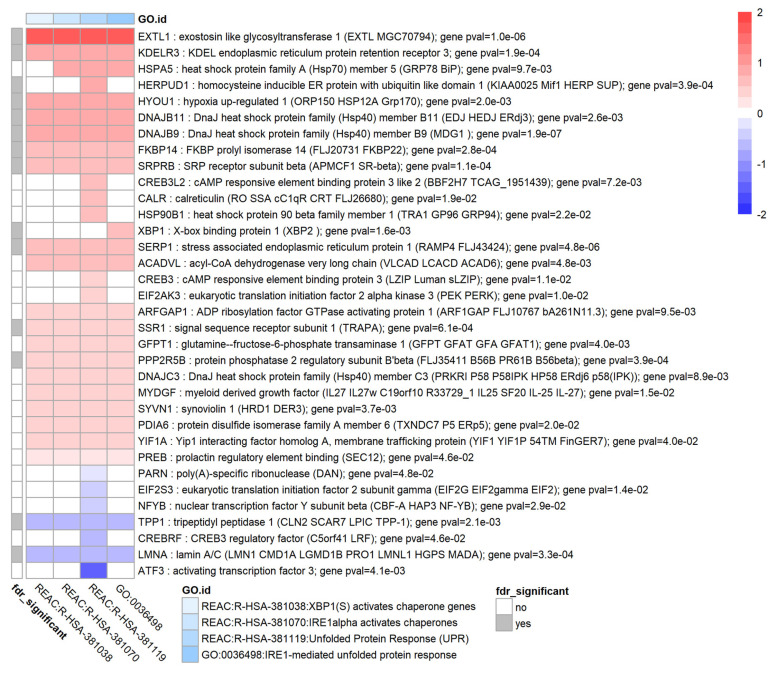
Enrichment results and genes associated with Unfolded Protein Response. Heatmap showing the genes with nominal *p*-value *p* < 0.05 for genes associated with the unfolded protein response (UPR). Heatmap colours correspond to fold change expressed as log_2_FC which has been limited to a range of [−2, 2] for clarity. The grey annotation bar on the left shows if the genome-wide false discovery rate FDR is smaller than 0.05 (in which FDR is the BH corrected *p*-value) for a particular gene. To further improve clarity, only 4 enriched terms within this cluster are shown.

Several enrichment results mapped to the cluster “unfolded protein response” such as Reactome pathways R-HSA-381038 and GO:0036498 BP-IRE1-mediated unfolded protein response. Differentially expressed genes with *p* < 0.05 within this cluster of enriched terms are shown in Figure 8. Some key UPR-related genes that were significantly upregulated (based on nominal *p*-values) were KDELR3, HSPA5 (also known as GRP78 or BiP), HSP90B1 (also known as GRP94 or TRA1), XBP1, and EIF2AK3 (also known as PERK). One key UPR gene that did not appear in this cluster was ERO1A (also known as ERO-1α) which was moderately upregulated (log_2_FC = 0.35, *p* = 0.02). 

### 3.10. TGF-β1 Increases Expression of Three XFS-Associated Genes from GWAS Studies

Using a recent list of GWAS genes known to be associated with XFS [36] it appeared that based on nominal *p*-values three XFS-related genes were upregulated due to TGF-β1 in normal HTM tissue namely LOXL1, POMP and RBMS3 (see Figure S2).

### 3.11. TGF-β1 May Modulate Retinoic Acid Signalling in HTM Cells

Retinoic acid signalling has been reported to play a role in XFG and XFS [12] but our enrichment analysis did not reveal any enrichment terms directly associated with retinoic acid signalling, metabolism or transport; although some genes that are important in retinoic acid pathways (such as STRA6) did appear in other enrichment terms such as “eye development” and “artery development”. To investigate retinoic acid pathways in our dataset, a custom gene list was created by combining gene lists of Reactome pathways R-HSA-5362517 Signaling by Retinoic Acid and GO:0001523 retinoid metabolic process resulting in a list of 118 unique genes in total. When considering genes with nominal *p* value < 0.05 it appeared that 6 out of these 118 genes were differentially expressed. Upregulated genes were STRA6 (log_2_FC = 2.3, *p* = 3.6 × 10^−6^), FABP5 and CYP1B1 and downregulated genes were ALDH1A1, AKR1C3, RDH5 and RXRA. In addition to the enrichment gene lists, RORB was downregulated (log_2_FC = −1.3, *p* = 8 × 10^−3^).

### 3.12. Validation of Differentially Expressed Genes by RT-qPCR

**Figure 9:**
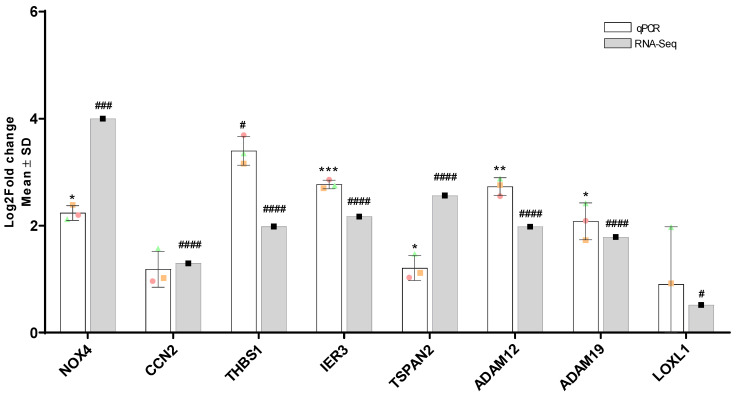
RT-qPCR confirmation and comparison of RNA-Seq results. A total of 3 control and TGF-β1 stimulated TM donor samples (labelled green, red, orange) that had previously been subjected to RNA-Seq analysis were used to validate gene expression patterns obtained from RNA-Seq analysis using RT-qPCR. Values were normalised to GAPDH and graphed as log_2_ fold change. Statistical significance was determined using a two-sample *t*-test (* *p* < 0.05, ** *p* < 0.005, *** *p* < 0.0005, ^#^ *p* < 0.05, ^###^ *p* < 0.0005, ^####^ *p* < 0.00005).

To validate the RNA-Seq results, several disease-relevant genes (LOXL1, NOX4, THBS1, CCN2, ADAM12, ADAM19) and high-ranking potentially interesting genes (IER3, TSPAN2) were selected for RT-qPCR validation (Figure 9) using the same samples that were previously used for RNA-Seq. Of these genes NOX4 (*p* = 0.02), THBS1 (*p* = 0.02), IER3 (*p* = 0.0007)), TSPAN2 (*p* = 0.02), ADAM12 (*p* = 0.03) and ADAM19 (*p* = 0.01) showed significant differential expression in the same direction as RNA-Seq. CCN2 and LOXL1 did not show significant differential expression. For LOXL1 a large biological variability could be observed which was also seen in the RNA-Seq results.

## 4. Discussion

In this study we performed a hypothesis-independent RNA sequencing analysis to investigate genome-wide alterations in the transcriptome of normal HTMs stimulated with TGF-β1 and investigate possible pathophysiological mechanisms driving XFG. Stimulation of normal HTM cells with TGF-β1 modulates pathways and biological processes that show much overlap with processes known to be associated with XFG such as ECM remodelling [38,39], modulation of oxidative stress [6], UPR [40] and changes in the expression of certain growth factors [14,41,42]. Based on these overlapping findings with XFG expression studies [4,18,43] the proposed cellular model could be a useful tool to gain more insight in the pathogenesis of XFG which is still poorly understood [1,2,3,44]. A comparison of relevant pathways and biological processes between this study and literature is discussed in more detail below.

Elastin (ELN) is a component of both XFM [16] and the ECM [45] and is a substrate for LOXL1. In keeping with our results ELN has been demonstrated to be upregulated by TGF-β1 in HTM cells [46]. Furthermore, ELN was upregulated in XFG patients [43] suggesting a possible causality between TGF-β1 and ELN expression in XFG. Additionally, fibrillin-1 (FBN1) is both an XFM [5,16] and ECM component which can be upregulated by TGF-β1 [47] and has been reported to be upregulated in XFG [43]. Certain laminin genes such as LAMC2 (γ-chain laminin) were upregulated in response to TGF-β1 which agrees with previous findings [47] while other α-chain laminins were downregulated. 

ADAM Metallopeptidase Domain 12 (ADAM12) and 19 (ADAM19) are involved in ECM remodelling and are upregulated in human glaucomatous lamina cibrosa cells [48]. In addition, ADAM12 was upregulated in response to TGF-β1 in HTM cells which agrees with our findings [47]; interestingly, ADAM19 is also a XFM component [4]. Matrix metalloproteinases 15 (MMP15) and 24 (MMP24) have not been reported to be upregulated in XFG nor in response to TGF-β1 treatment to our knowledge [49] but were nevertheless upregulated in our results. However, similar MMP family members such as MMP2 [38] have been reported to be upregulated but not activated. Similarly, TIMP metallopeptidase Inhibitors (TIMP1, TIMP2, TIMP3, TIMP4) were not differentially expressed in our experiment while these have been reported to be upregulated in the AH of individuals with XFS [38]. Serpin family E Member 1 (SERPINE1) which reduces MMP activity, was increased in our data and has also been reported to be increased in the aqueous humour of patients with glaucoma [39]. Furthermore, SERPINE1 has been reported to be upregulated in response to TGF-β1 [50]. Secreted Protein Acidic Additionally, Cysteine Rich (SPARC) also known as osteonectin is a matricellular protein and increased expression is associated with increased IOP [51]. Similar to our findings SPARC has been demonstrated to be increased by TGF-β2 [52]. Tetraspanin 2 (TSPAN2) has been reported to be expressed by fibroblasts and involved in ECM organisation [53]. Furthermore, it has been reported that expression of TSPAN2 in vascular smooth muscle tissue was upregulated due to TGF-β1 treatment which agrees with our findings in HTM cells [54]. Several collagen species such as COL1A1, COL4A1, COL4A2 and COL5A1 have previously been shown to be upregulated in response to TGF-β1 treatment in HTM tissue [47,55] which agrees with our findings. In contrast, several studies found decreased expression of several collagen types in other ocular tissues obtained from XFS patients. In lens capsular epithelium, COL4A1 and COL4A1 were shown to be decreased [4] while in lamina cribrosa tissue no change in expression of COL4A1 was found between XFS patients and controls suggesting that differences in the expression of collagen genes may be tissue-specific or dependent on disease progression.

**Figure 10:**
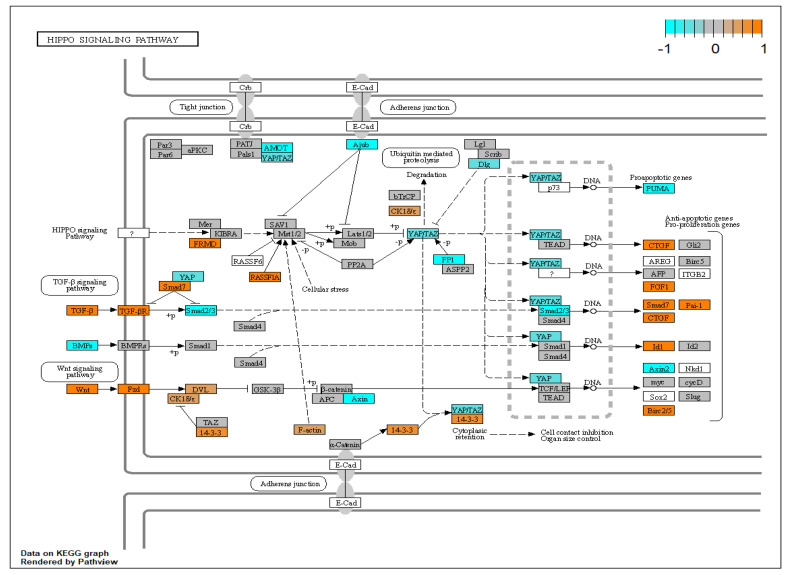
Annotation of genes in the KEGG generated hsa04390 pathway; the Hippo signaling pathway. Differentially expressed genes in the hsa04390 pathway have been annotated using R package Pathview. Gene expression has been normalised to a range of [−1, 1] to enhance visibility in which orange corresponds to upregulation while cyan corresponds to downregulation. Grey coloured genes are expressed but not differentially so. White coloured genes were not found to be expressed in our dataset.

XFG is associated with changes in growth factor expression. In agreement with our findings CCN2 (also known as CTGF) has been reported to be elevated in the aqueous humour of XFS patients with respect to controls [41,56]. Furthermore, it has been reported that TGF-β1 does not only upregulate expression of CCN2 but CCN2 can also cause upregulation of TGF-β1 which points to a self-sustaining positive feedback loop motif [57]. Indeed, also TGF-β1 was upregulated in our experiment (log_2_FC = 0.60, *p* = 0.05) which may be in part due to CCN2. Vascular Endothelial Growth Factor A (VEGFA) promotes proliferation of vascular endothelial cells and is upregulated in XFG [42] which agrees with our findings. Furthermore, VEGFA has been shown to be upregulated by TGF-β2 [58]. Nerve growth factor (NGF) has been reported to be expressed by HTM cells [59] and was upregulated by TGF-β in chondrocytes [60]. Similar to our findings, thrombospondin-1 (THBS1) has previously been shown to be upregulated by TGF-β1 in TM cells and also to be upregulated in POAG [61]. THBS1 is capable of activating latent TGF-β1 by inducing conformal changes in the large latent complex [62] implying another potential positive feedback loop. Gremlins (GREM1 and GREM2) have been demonstrated to indirectly activate the canonical TGF-β pathway in HTM cells by blocking inactivation of this pathway [63] and are upregulated in glaucomatous trabecular meshwork tissue [64]. In renal cells, TGF-β1 induces gremlin expression [65] which agrees with our findings in HTM cells. With respect to interleukins and cytokines, interleukin 11 (IL-11) is increased in the AH in POAG [66] and upregulated in other fibrotic diseases such as idiopathic pulmonary fibrosis and furthermore administration of IL-11 can induce lung fibrosis [67]. TGF-β1 has been shown to induce expression of IL-11 in gingival fibroblasts [68]. Interleukin 12 has been shown to be increased in the AH of POAG patients [69] but has also been reported to be lower in XFG with respect to POAG [70]. The canonical Wnt pathway in HTM cells has been implicated in regulation of IOP [71] and Wnt signalling was also enriched in our results. Several Wnt Family members such as WNT5A and WNT7B play a role in fibrotic diseases such as idiopathic pulmonary fibrosis [72] and renal fibrosis [73]. Furthermore, WNT5A and WNT11 have been shown to be regulated by TGF-β1 in human lung fibroblasts [72]. Interestingly a mouse model in which the eyes were injected with adenovirus expressing WNT5A showed XFG-like features such as accumulation of deposits [74]. Dickkopf WNT Signalling Pathway Inhibitor 1 (DKK1) which is a Wnt pathway inhibitor, has been shown to increase IOP in a mouse model upon intravitreal injection of a viral vector expressing DKK1 [75]. To demonstrate the interplay between the TGFβ and Wnt pathways in a graphical way, R package Pathview [35] was used to annotate genes in the KEGG “hippo signaling pathway” hsa04390 [76] (Figure 10). This graph shows upregulation of the TGFβ and Wnt pathway and also clearly demonstrates the SMAD7 negative feedback loop in the canonical TGFβ pathway and upregulation of CCN2(CTGF), FGF1 and SERPINE1 (PAI-1).

Increased oxidative stress and reduced antioxidant capacity are both associated with XFG [9,77] and oxidative stress is an enriched process in our results. Increased oxidative stress can lead to activation of TGF-β1 via several different mechanisms such as conformal change of the LAP-TGFβ complex [78] or activation of matrix metalloproteinases [79]. Interestingly, direct liberation of TGF-β from the LAP-TGFβ complex by reactive oxygen species has only been demonstrated for TGF-β1 and not for the other two TGF-β isoforms [78]; so perhaps this particular mechanism plays a larger role in the pathogenesis of XFG in which TGF-β1 is the predominant TGF-β isoform. Our results demonstrated a decrease in SOD2 expression in response to TGF-β1 which agrees with an observed decrease in SOD2 expression in response to TGF-β in smooth muscle cells [80]. In this latter study the decrease in SOD2 could partially be prevented by silencing NOX4 using siRNA. In contrast, it has been reported that enzymatic activity [81], concentration in AH [82] and expression in ciliary processes [17] of SOD2 was increased in XFG patients compared to cataract controls. Possibly there is a transient effect depending on stage of the disease or other factors besides TGF-β1 play a role here. Our results also showed a decrease in SOD3 while this enzyme was increased in lens capsular epithelium of XFG patients [4] which may be explained by tissue-dependence or time-dependence. In XFG there is less availability of glutathione (GSH) in a reduced state when compared to controls [81]. In agreement with this observation, our results showed an increase in several glutathione peroxidases (GPX3, GPX7, GPX8) and reduction of glutathione reductase (GSR) which would also lead to less reduced GSH. It is interesting to note that decreased expression of GSR in response to TGF-β1 was also observed in human kidney tubular epithelial (HK2) cells [83]. In addition, decreased expression of glutathione S-transferase is associated with XFG [17,18] which agrees with downregulation of several GST genes in our results. Another mechanism by which available GSH can be lowered is by reduced synthesis due to downregulation of glutamate-cysteine ligase catalytic subunit (GCLC). GCLC has been reported to be lowered by TGF-β1 [22,84]. Our results showed a trend for decreased expression of GCLC but with insufficient statistical power. Peroxidasin (PXDN) is involved in crosslinking collagen IV while utilising hydrogen peroxide, so it has some anti-oxidant capability [85]. Interestingly it has been associated with Nrf2 and was reported to be increased (similar to our results) in kidney fibrosis [86]. Noteworthy is that certain PXDN variants have been associated with a congenital form of glaucoma [87]. TGF-β1 and TGF-β2 have been shown to upregulate expression of NADPH oxidase 4 (NOX4) in several ocular tissues [88,89,90] which agrees with our findings. Upregulation of NOX4 further increases oxidative stress due to production of hydrogen peroxide which may switch on redox signalling pathways and is also required to crosslink collagen IV by PXDN, as described above. 

**Figure 11:**
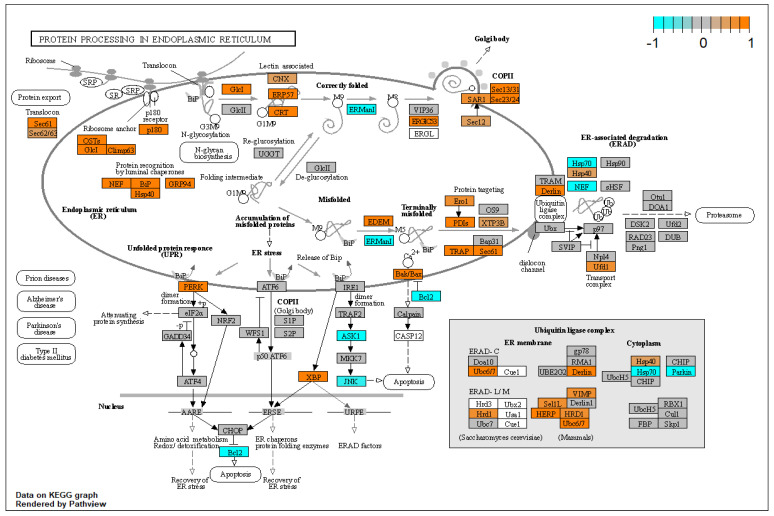
Annotation of differentially expressed genes in KEGG hsa04141 pathway. Differentially expressed genes in the hsa04141 pathway (protein processing in endoplasmic reticulum) have been annotated using R package pathview. Gene expression has been normalised to a range of [−1, 1] to enhance visibility in which orange corresponds to upregulation while cyan corresponds to downregulation. Grey coloured genes are expressed but not differentially so. White coloured genes were not found to be expressed in our dataset.

The unfolded protein response (UPR) is induced in certain cases of endoplasmic reticulum (ER) stress such as increased protein synthesis or the presence of misfolded proteins which may be caused by protein oxidation and other mechanisms [40,91]. The UPR consists of three parallel pathways via EIF2AK3 (PERK), ATF6 and IRE1α [91] that are activated via HSPA5 (GRP78, BIP) which acts as a sensor of ER stress. In glaucomatous TM (GTM) cells the UPR is chronically activated which may point to a failure in regaining endoplasmic homeostasis [23]. This state of chronic activation can lead to activation of an apoptotic pathway resulting in cell death, and furthermore to increased oxidative stress via activation of CHOP, because CHOP activates endoplasmic reticulum oxidoreductase 1α ERO1A [23]. Protein oxidation itself may lead to an increase in protein misfolding rate which then further increases ER stress, so this points to a positive feedback mechanism. Our results indicate that TGF-β1 activates the UPR in primary HTM cells. Key genes associated with UPR such as HSPA5 (GRP78, BiP), HSP90B1 (GRP94) and EIF2AK3 (PERK) all are significantly upregulated based on a per-gene *p*-value < 0.05, while several other UPR genes, such as XBP1, were also significantly upregulated on a genome-wide scale (FDR < 0.05). The expression of DDIT3 (CHOP) and EIF2A was unchanged. Interestingly ERO1A, a UPR gene that seems to be more involved in a chronic ER stress state as outlined above, was significantly upregulated which could contribute to further protein misfolding, increased oxidative stress and apoptosis of TM cells. To further elucidate changes in the UPR due to TGF-β1, the expression of all genes in KEGG pathway hsa04141 “protein processing in the endoplasmic reticulum” [76] was visualised using R visualisation package Pathview [35]. This annotated pathway clearly shows that ER stress is present and that the UPR is activated in our experiment (Figure 11). This cellular model of XFG may support the development of novel therapeutic interventions targeting the UPR and ER stress [92,93,94]. 

Of the GWAS genes known to be associated with XFG [36], LOXL1, RBMS3 and POMP were all upregulated in response to TGF-β1 in our experiments. LOXL1 (Lysyl Oxidase Like 1) plays a role in the production of connective tissue by crosslinking collagen and elastin and furthermore LOXL1 is a component of exfoliation material [5]. LOXL1 has been demonstrated to be upregulated due to TGF-β1 in Tenon’s ocular fibroblasts [95]. In contrast, LOXL1 was downregulated in human Tenon’s ocular fibroblasts from XFG patients compared to controls while the LOXL1 concentration in aqueous humour was increased in XFG patients [96]. This early upregulation of TGF-β1 is not unexpected as LOXL1 expression was increased at an early stage of XFS and decreased at a later stage [97]. Therefore, our results showed an increase in LOXL1 expression in response to TGF-β1 which may correlate with an early stage of XFS. Possibly, longer exposure of normal HTM cells to TGF-β1 may alter LOXL1 expression. RBMS3 (RNA Binding Motif Single Stranded Interacting Protein 3) is an RNA-binding protein which controls gene expression by binding to the 3′ UTR of mRNA thus stabilising and increasing half-life of these transcripts. RBMS3 has been reported to bind to PRRX1 [98,99] and PRRX1 is associated with fibrosis and epithelial-to-mesenchymal transition both of which are relevant processes in both POAG and XFG. Furthermore, RBMS3 has been reported to stabilise several members of the SMAD family such as SMAD2 in zebrafish [100] and SMAD2, SMAD3 and SMAD4 in triple-negative breast cancer cells [101]. These specific SMAD members are part of the canonical TGF-β pathway and increased expression by increased half-life would enhance activation of this pathway. Therefore, it is likely that the increased RBMS3 expression shown in this study may contribute to several pro-fibrotic biological processes. POMP (Proteasome Maturation Protein) is a chaperone for proteasome assembly. It has been reported that POMP protein is downregulated in iris and ciliary body tissue obtained from XFG patients when compared to age-matched controls [36]. In contrast, it was reported that POMP was upregulated in lens capsular epithelial cells from XFG patients [4] which would agree with our results.

Retinoic acid signalling has been reported to play a role in the pathogenesis of XFG and to modulate the activated TGF-β pathway by changing levels of phosphorylation [12]. Our results showed differential expression of several genes involved in retinoic acid transport and signalling. Transport protein STRA6 was upregulated in our results which differs from the downregulation previously reported in XFG patients [12]. It should be noted that this gene did show large biological variation between donors. It would be interesting to investigate expression of STRA6 in response to TGF-β1 in a greater number of biological replicates and at additional time-points to determine if there may be any transient effects in its expression. The expression of ALDH1A1 and RORB was reduced which agrees with observed reduced expression in XFG patients [12]. Furthermore, the expression of retinal reductase AKR1C3 (reducing retinal back to retinol) and reductase RDH5 (oxidation of 11-cis-retinol to 11-cis-retinal) were decreased while the expression of cytochrome p450 gene CYP1B1 was increased. Overall, there does seem to be an effect of the TGF-β pathway on retinoic acid metabolism but the overall effect requires further research to clarify.

A comparison between findings in this study and some findings reported in the extensive XFG literature is shown in Table 4 where it should be mentioned that the reported findings from literature are by no means complete. From this comparison it is clear that there is significant overlap between pathways and biological processes that are known to play a role in XFG and those perturbed by TGF-β1 in normal HTM tissue indicating that the proposed model could be valuable to study several aspects of the pathogenesis of XFG and XFS. 

## 5. Conclusions

Pseudoexfoliation glaucoma (XFG) is an aggressive form of secondary open angle glaucoma and activation of the TGF-β pathway has been implicated in its pathogenesis. Herein, we report genome-wide transcriptional changes identified in normal human trabecular meshwork (HTM) cells in response to TGF-β1 stimulation. Our results identified multiple differentially expressed genes including several genes known to be present in exfoliation material. Significantly altered pathways, biological processes and molecular functions included extracellular matrix remodelling, Hippo and Wnt pathways, the unfolded protein response, oxidative stress, and the antioxidant system. These dysregulated pathways offer the potential to develop novel therapeutic interventions in XFG. Hippo signalling is a therapeutic target in cancer biology and inhibitors of YAP/TAZ, the downstream effectors of the Hippo pathway are attractive targets [103,104]. The inhibition of YAP with verteporfin in lamina cribrosa cells showed therapeutic benefits [105]. ER stress and the unfolded protein response (UPR) have also gained interest in glaucoma and ophthalmology as therapeutic targets [106,107,108]. There are a number of potentially druggable stress pathways [107] and genetic manipulation or pharmacological inhibition of the ATF4–CHOP–GADD34 pathway has shown therapeutic benefit in the trabecular meshwork in murine and human glaucoma [108,109].This cellular model of pseudoexfoliation glaucoma cannot capture all features of this complex disease, but can provide some insight into disease pathogenesis and support the development of novel therapeutic interventions.

## Figures and Tables

**Table 1:** Top 50 differentially expressed genes, ranked by false discovery rate (FDR). For each gene the current and previously used symbols, name, log_2_FC (binary logarithm of fold change), *p*-value and adjusted *p*-value in the form of false discovery rate (FDR) are shown.

Gene Symbol	Gene Name	log_2_FC	*p*-Value	FDR
IER3	immediate early response 3	2.17	1.05 × 10^−42^	1.36 × 10^−38^
DSP	desmoplakin	1.36	7.75 × 10^−31^	4.99 × 10^−27^
TSPAN2	tetraspanin 2	2.59	1.90 × 10^−23^	8.16 × 10^−20^
ADAM12	ADAM metallopeptidase domain 12	1.98	1.80 × 10^−20^	5.80 × 10^−17^
TP53I3	tumor protein p53 inducible protein 3	1.24	1.14 × 10^−17^	2.94 × 10^−14^
P4HA2	prolyl 4-hydroxylase subunit alpha 2	1.76	2.14 × 10^−17^	4.59 × 10^−14^
DCLK1 (DCAMKL1)	doublecortin like kinase 1	–2.10	3.47 × 10^−17^	6.39 × 10^−14^
GREM1 (CKTSF1B1)	gremlin 1, DAN family BMP antagonist	3.08	1.42 × 10^−16^	2.29 × 10^−13^
PCDH10	protocadherin 10	2.10	3.02 × 10^−16^	4.32 × 10^−13^
ATP10A (ATP10C)	ATPase phospholipid transporting 10A	2.13	1.03 × 10^−15^	1.33 × 10^−12^
SEMA7A (SEMAL)	semaphorin 7A	2.65	2.78 × 10^−15^	3.26 × 10^−12^
FSTL3	follistatin like 3	2.43	1.43 × 10^−14^	1.54 × 10^−11^
SPHK1	sphingosine kinase 1	1.41	6.41 × 10^−14^	6.35 × 10^−11^
TGFBI	transforming growth factor beta induced	2.48	4.71 × 10^−13^	3.79 × 10^−10^
LTBP2 (LTBP3 C14orf141)	latent transforming growth factor beta binding protein 2	1.81	4.45 × 10^−13^	3.79 × 10^−10^
CADM1 (TSLC1 IGSF4)	cell adhesion molecule 1	1.68	4.40 × 10^−13^	3.79 × 10^−10^
PLPP4 (PPAPDC1)	phospholipid phosphatase 4	1.33	6.69 × 10^−12^	5.07 × 10^−9^
TLL2	tolloid like 2	1.88	7.94 × 10^−12^	5.69 × 10^−9^
AMIGO2	adhesion molecule with Ig like domain 2	1.85	9.79 × 10^−12^	6.64 × 10^−9^
SERPINE2 (PI7)	serpin family E member 2	2.16	1.35 × 10^−11^	8.31 × 10^−9^
ALDH6A1 (MMSDH)	aldehyde dehydrogenase 6 family member A1	–1.22	1.33 × 10^−11^	8.31 × 10^−9^
ODAPH (C4orf26)	odontogenesis associated phosphoprotein	10.70	1.87 × 10^−11^	1.07 × 10^−8^
NOG (SYNS1 SYM1)	noggin	1.97	1.91 × 10^−11^	1.07 × 10^−8^
RCAN2 (DSCR1L1)	regulator of calcineurin 2	–1.04	2.71 × 10^−11^	1.46 × 10^−8^
RN7SL1 (RN7SL)	RNA component of signal recognition particle 7SL1	–1.08	6.59 × 10^−11^	3.39 × 10^−8^
MYOZ1 (MYOZ)	myozenin 1	3.47	1.03 × 10^−10^	5.12 × 10^−8^
ESM1	endothelial cell specific molecule 1	4.29	1.21 × 10^−10^	5.76 × 10^−8^
MME	membrane metalloendopeptidase	–2.49	1.53 × 10^−10^	7.03 × 10^−8^
PMEPA1 (TMEPAI)	prostate transmembrane protein, androgen induced 1	2.50	2.85 × 10^−10^	1.22 × 10^−7^
COL4A1	collagen type IV alpha 1 chain	1.89	2.76 × 10^−10^	1.22 × 10^−7^
SPARC (ON)	secreted protein acidic and cysteine rich	1.38	3.34 × 10^−10^	1.39 × 10^−7^
GALNT10	polypeptide N-acetylgalactosaminyltransferase 10	1.11	3.98 × 10^−10^	1.60 × 10^−7^
ACTC1 (ACTC)	actin alpha cardiac muscle 1	3.62	5.83 × 10^−10^	2.28 × 10^−7^
TSPAN13 (TM4SF13)	tetraspanin 13	2.29	7.79 × 10^−10^	2.95 × 10^−7^
EPHB2 (DRT EPHT3)	EPH receptor B2	1.48	9.65 × 10^−10^	3.55 × 10^−7^
LIMK2	LIM domain kinase 2	1.01	1.03 × 10^−9^	3.68 × 10^−7^
A2M	Alpha-2-macroglobulin	–2.41	1.06 × 10^−9^	3.68 × 10^−7^
SVIL	supervillin	–1.46	2.75 × 10^−9^	9.33 × 10^−7^
CSRP2	cysteine and glycine rich protein 2	0.99	3.19 × 10^−9^	1.05 × 10^−6^
PCDH9	protocadherin 9	1.00	3.60 × 10^−9^	1.16 × 10^−6^
CDH2 (NCAD)	cadherin 2	1.88	3.84 × 10^−9^	1.21 × 10^−6^
S1PR3 (EDG3)	sphingosine-1-phosphate receptor 3	–1.27	4.15 × 10^−9^	1.27 × 10^−6^
MT1L (MT1)	metallothionein 1L, pseudogene	1.60	4.25 × 10^−9^	1.27 × 10^−6^
NNMT	nicotinamide N-methyltransferase	1.20	4.51 × 10^−9^	1.32 × 10^−6^
THBD	thrombomodulin	1.81	7.09 × 10^−9^	2.03 × 10^−6^
MOXD1	monooxygenase DBH like 1	–1.26	8.46 × 10^−9^	2.37 × 10^−6^
TMEM87B	transmembrane protein 87B	1.71	9.06 × 10^−9^	2.48 × 10^−6^
GPRC5B	G protein-coupled receptor class C group 5 member B	1.41	1.21 × 10^−8^	3.19 × 10^−6^
EPGN	epithelial mitogen	1.22	1.21 × 10^−8^	3.19 × 10^−6^
VDR	vitamin D receptor	1.30	1.80 × 10^−8^	4.64 × 10^−6^

**Table 2:** Top 40 enrichment results from KEGG, reactome and GO biological processes and molecular functions ranked by false discovery rate (FDR). BP = Biological Processes and MF = Molecular Function where pval indicates the nominal *p*-value for each enrichment result.

Enrichment Result	*p* Value
BP-extracellular matrix organization (GO:0030198)	3.34 × 10^−22^
BP-extracellular structure organization (GO:0043062)	5.75 × 10^−16^
BP-external encapsulating structure organization (GO:0045229)	4.04 × 10^−15^
Extracellular matrix organization Homo sapiens (REAC:R-HAS-1474244)	3.11 × 10^−14^
BP-collagen fibril organization (GO:0030199)	1.80 × 10^−11^
Elastic fibre formation Homo sapiens (REAC:R-HSA-1566948)	8.02 × 10^−9^
BP-supramolecular fiber organization (GO:0097435)	1.95 × 10^−8^
XBP1(S) activates chaperone genes Homo sapiens (REAC:R-HSA-381038)	2.03 × 10^−8^
BP-IRE1-mediated unfolded protein response (GO:0036498)	2.03 × 10^−8^
Collagen formation Homo sapiens (REAC:R-HSA-1474290)	2.85 × 10^−8^
IRE1alpha activates chaperones Homo sapiens (REAC:R-HSA-381070)	3.29 × 10^−8^
MF-receptor ligand activity (GO:0048018)	9.49 × 10^−8^
BP-negative regulation of pathway-restricted SMAD protein phosphor. (GO:0060394)	1.26 × 10^−7^
Collagen biosynthesis and modifying enzymes Homo sapiens (REAC:R-HSA-1650814)	1.84 × 10^−7^
BP-regulation of cell population proliferation (GO:0042127)	4.90 × 10^−7^
Unfolded Protein Response (UPR) Homo sapiens (REAC:R-HSA-381119)	1.33 × 10^−6^
BP-ventricular cardiac muscle tissue morphogenesis (GO:0055010)	1.57 × 10^−6^
BP-regulation of pathway-restricted SMAD protein phosphorylation (GO:0060393)	3.90 × 10^−6^
Dilated cardiomyopathy—Homo sapiens (human) (KEGG:hsa05414)	5.11 × 10^−6^
MF-growth factor activity (GO:0008083)	8.42 × 10^−6^
BP-negative regulation of cell population proliferation (GO:0008285)	8.55 × 10^−6^
BP-positive regulation of cell population proliferation (GO:0008284)	8.70 × 10^−6^
BP-actomyosin structure organization (GO:0031032)	1.05 × 10^−5^
BP-positive regulation of cell differentiation (GO:0045597)	1.09 × 10^−5^
Glycosaminoglycan biosynthesis—heparan sulfate/heparin—(KEGG:hsa00534)	1.13 × 10^−5^
Arrhythmogenic right ventricular cardiomyopathy—(KEGG:hsa05412)	1.20 × 10^−5^
Hypertrophic cardiomyopathy—Homo sapiens (human) (KEGG:hsa05410)	1.23 × 10^−5^
Assembly of collagen fibrils and other multimeric structures (REAC:R-HSA-2022090)	1.34 × 10^−5^
BP-heart development (GO:0007507)	1.50 × 10^−5^
ECM proteoglycans Homo sapiens (REAC:R-HSA-3000178)	1.59 × 10^−5^
Integrin cell surface interactions Homo sapiens (REAC:R-HSA-216083)	1.66 × 10^−5^
BP-positive regulation of non-canonical Wnt signaling pathway (GO:2000052)	1.86 × 10^−5^
BP-blood vessel morphogenesis (GO:0048514)	1.87 × 10^−5^
BP-ventricular compact myocardium morphogenesis (GO:0003223)	1.98 × 10^−5^
L1CAM interactions Homo sapiens (REAC:R-HSA-373760)	2.50 × 10^−5^
BP-regulation of angiogenesis (GO:0045765)	2.54 × 10^−5^
BP-positive regulation of cellular process (GO:0048522)	3.09 × 10^−5^
MF-vascular endothelial growth factor receptor binding (GO:0005172)	3.10 × 10^−5^
BP-ventricular septum morphogenesis (GO:0060412)	3.51 × 10^−5^
BP-regulation of cell migration (GO:0030334)	3.60 × 10^−5^

**Table 3:** Enrichment clusters after clustering of enrichment terms. Shown is the cluster name, number of enrichment terms within the cluster (column #) and top 10 genes ranked by FDR.

Cluster Name	#	Top 10 Genes Ranked by FDR
fibroblast proliferation; G1/S	24	GREM1,SEMA7A,FSTL3,SPHK1,TGFBI,SERPINE2,NOG,ESM1,EPHB2,S1PR3
ECM remodelling	23	DSP,ADAM12,P4HA2,GREM1,SEMA7A,TGFBI,LTBP2,TLL2,MYOZ1,COL4A1
morphogenesis; wound healing	16	DSP,NOG,MYOZ1,COL4A1,ACTC1,CDH2,DYSF,NRG1,TPM1,PDLIM7
AGE-RAGE;hippo;focal adhesion	15	COL4A1,THBD,DYSF,KCNK3,COL4A2,NOX4,GADD45B,TPM1,LEFTY2,ID3
coagulation; complement; NO	13	SERPINE2,MYOZ1,SPARC,EPHB2,A2M,THBD,APOE,LEFTY2,C1R,PDE5A
TGFb and BMP pathway	9	GREM1,FSTL3,NOG,PMEPA1,EPHB2,RASSF2,LEFTY2,IGFBP3,ID3,SKIL
unfolded protein response	8	FSTL3,SPHK1,TGFBI,MME,GALNT10,CDH2,VDR,APOE,SPON2,HSPA2
protein phosphorylation	7	GREM1,SPHK1,NOG,PMEPA1,LIMK2,NNMT,RASSF2,LEFTY2,ANKLE2,IGFBP3
cell migration; angiogenesis	7	ADAM12,GREM1,SPHK1,ESM1,SPARC,APOE,COL4A2,ANGPTL4,E2F7,AMOT
RHO GTPase; L1CAM	7	DSP,SEMA7A,COL4A1,EPHB2,LIMK2,CDH2,COL4A2,ANGPTL4,EPHB6,LEFTY2
epithelial to mesenchymal trans.	6	DCLK1,GREM1,CADM1,ODAPH,NOG,EPHB2,CDH2,VDR,APOE,NRG1
cell motility	6	SEMA7A,SPHK1,SERPINE2,NOG,EPHB2,CDH2,NRG1,TPM1,IGFBP3,AMOT
glycosaminoglycan synthesis	5	XYLT1,CSGALNACT2, NDST1, B4GALT1, EXTL1, HS3ST2, HS3ST3B1, HAS1
branched chain amino acid catab.	4	ALDH6A1,ALDH3A2,PPM1K,OXCT1,HIBADH,MCCC2,ALDH7A1,ACADSB
endopeptidase inhibition	4	SERPINE2,A2M,FURIN,ATP13A2,SERPINE1,PROS1,PLAUR,NGF,AGT,RECK
eye morphogenesis	4	VEGFA,STRA6,COL5A1,FBN1,SALL2,LAMA1,COL5A2,SMOC1,FBN2,PBX1
neuron differentiation	4	GREM1,CDH2,GPRC5B,NREP,IGFBP3,DDR2,DKK1,SCIN,PPARG,PTN
response to growth factor	3	SPHK1,LTBP2,GAS1,LEFTY2,VEGFA,FURIN,ITGB5,BMP4,TGFBR3,HAS1
cardiomyopathy	3	DSP,ACTC1,CDH2,TPM1,SGCG,LAMA1,ITGB5,ITGA5,ITGAV,TPM2
gland development	3	NRG1,E2F7,STRA6,TGFBR3,TGFBR1,CITED2,XBP1,PKD2,TG
myofibril assembly	3	MYOZ1,ACTC1,CSRP2,TPM1,LMOD1,EPB41L2,ITGB5,CDC42BPA,ANKRD1,MYH10
pos.regulation of Wnt pathway	3	DKK1,WNT5B,SFRP1,PLEKHA4, RSPO3
apoptosis	3	TP53I3, GREM1,SPHK1,RASSF2,GAS1,ANGPTL4,GADD45B,ANKLE2, IGFBP3
kidney/lung development	3	MME,VEGFA,STRA6,FOXD1,BMP4,TGFBR1,KANK2,WNT7B,AGT,CTSH
aldehyde dehydrogenase activity	2	P4HA2,P4HA1,ALDH1L2,ALDH3A2,ALDH3B1,SAT1,PYCR1,SRM,ALDH7A1
cGMP signalling	2	APOE,IRAG1,PDE3A,HTR2B,PDE7B,AQP1,AKAP6
nitric oxide signalling	2	SPHK1,RCAN2,APOE,FPR1,CHRM3,HTR2B,AGT,LAT2,GUCY1A1
oxidative stress	2	NOX4,TPM1,ALDH3B1,ATP13A2,SOD3,GPX3,PYCR1,GPX8,CPEB2,PPARGC1A
T cell mediated cytotoxicity	2	CADM1,EMP2,MICA,CTSH
regulation signal transduction	1	ESM1,TPD52L1,TGFBR1,EMP2,ERFE,ITGA5,ITGAV,MYORG,CITED2,SFRP1
actinin binding	1	CSRP2,PDLIM4,PPARG,CSRP1,PKD2
lipid storage	1	PPARG,ITGAV,TTC39B,ITGB3
Ras protein transduction	1	EPHB2,EPS8,ARHGEF2,KANK2,NGF,RAPGEF1,OPHN1,DENND4C,LRRC59
regulation of cell–cell adhesion	1	FSTL3,ADAM19,PLPP3,PIEZO1,CITED2,PLAUR,SMAD7
endothelial cell migration	1	VEGFA,EMP2,DPP4,PLEKHG5,LOXL2,ID1
bleb assembly	1	EMP1,EMP2,P2RX7
actin binding	1	MYOZ1,TPM1,PDLIM7,PDLIM4,SCIN,EPS8,SPTBN5,UTRN,TPM2,CALD1
striated muscle cell differentiation	1	NRG1,HDAC5,HDAC9
lipoprotein transport	1	PPARG,ZDHHC17,VMP1
renal tubule development	1	COL4A1,PKD2,MTSS1
regulation of cell migration	1	VEGFA,CARD10,HDAC5,PTGS2,HDAC9
blood circulation	1	ACTC1,SGCG,ELN,HTR7,SERPING1,TBC1D8,GUCY1A1
protein kinase C binding	1	DSP,CAVIN2,HDAC5,ITGAV,PKP2,HDAC9,IRS1
muscle tissue development	1	CSRP2,STRA6,BMP4,CSRP1
potassium channel activity	1	KCNK3,GRIK2,KCNK6,KCNMB1,KCNT2,KCNC4,AQP1,KCNA1,PKD2,KCND1
bone resorption	1	ACP5,RAB3D,TPP1
natural killer cell differentiation	1	TOX,PBX1,NFIL3
amyloid precursor catabolism	1	APOE,FLOT2,PICALM,BIN1
phagosome	1	C1R,DYNC2H1,ITGB5,ATP6V1G1,SEC61G,ITGA5,ITGAV,ITGA2,ITGB3,THBS2
limb development	1	GREM1,NOG,DKK1,MAP3K20,SMOC1
detection of a mechanical stimulus	1	CDH2,PIEZO1,PKD1L2,PKD2
muscle cell differentiation	1	DKK1,HDAC5,XBP1,FZD7
long term synaptic potentiation	1	EPHB2,APOE,CALB2,PTN,LGMN
chordate embryonic development	1	NOG,XYLT1,VEGFA,TGFBR1,ARNT2,WNT7B,PKD2
cellular response to hypoxia	1	VEGFA,PTGS2,LMNA,SFRP1,CPEB2,SCN2A,TBL2,AQP1,HYOU1

**Table 4:** Comparison of pathways and biological processes with literature. This table compares pathways and gene ontologies reported to be relevant in the pathophysiology of XFG and those found in this work. ↑ indicates upregulation, ↓ downregulation, == no change, ? unknown. Important alias gene symbols have been mentioned between brackets.

Pathway/Gene Ontology	This Work	Reported in XFG Literature
exfoliation material XFM gene expression	VCAN, ELN, LTBP2, ADAM19, LDHA, FBN1, MYL6, LOXL1, MYH9 ↑	LTBP2, APOE, IRAG1, ADAM19, LDHA, FBN1 ↑ [5], LAMA1, ELN ↑ [5], VCAN, VTN, C4B, TKT, BFSP1, LOXL1, MYL6, MYH9, C4A, S100A6, LAMB1, SASH1, HSPB1, YWHAB, PKM, ENO1, TRRAP, FN1, CLU, ADAM21, ANXA7, FBLN2, SDC3, CRYAB, HEATR1, ARHGAP42, LAMC1, COL18A1, C3, LTBP1, PRDX2, NID1, TIMP3, RASD1, EMILIN1, VIM, FTH1, ANXA1, MFAP2, SDC2, ALDH3A1
	APOE, LAMA1, C4B, VTN, IRAG1, C4A, TKT, BFSP1, S100A6 ↓	
XFS GWAS genes	LOLX1 ↑ POMP ↑ RBMS3 ↑	LOXL1 ↑ and ↓, may be a temporal effect [97] POMP ↑ [4] and ↓ [36]
extracellular signal molecules	CCN2 (CTGF) ↑	CCN2 ↑ [41]
	TGF-β1 ↑	TGF-β1 ↑ [14]
	THBS1 ↑, THBS2 ↓	THBS1 ↑ in POAG [61]
	EDN1 ↑	EDN1 in POAG ↑ [102]
	VEGFA ↑	VEGFA ↑ [42]
interleukins, complement	IL-6, IL-8 ==	IL-6↑ IL-8 ↑
	IL11↑ IL12A ↑	IL-11, IL-12 ↑ in POAG [69,70]
TGF-β pathway regulators	GREM1,2 ↑	GREM1,2 ↑ [64]
Wnt pathway	WNT5B ↑ WNT2B ↓ WNT7B ↑ DKK1 ↑	Wnt pathway implicated [74]
unfolded protein response	UPR pathway ↑ KDELR3 ↑ HSPA5 (GRP78) ↑ HSP90B1 (TRA1) ↑ XBP1 ↑ EIF2AK3 ↑ ERO1A ↑	UPR pathway implicated [40]
oxidative stress & antioxidant system	GPX3 ↑, GPX7 ↑, GPX8 ↑, GSR ↓ SOD2 ↓, SOD3 ↓ GSTM4 ↓ GSTM3 ↓ GSTA4 ↓ NOX4 ↑	SOD2 ↑ [17] SOD3 ↑ [4] GST1 ↓ [18] NOX4 ?
retinoic acid signalling	STRA6 ↑ ALDH1A1 ↓ RORB ↓ AKR1C3 ↓ RDH5 ↓	STRA ↓ [12] ALDH1A1↓ RORB ↓ [12] AKR1C3 ? RDH5 ?
ECM remodelling	TIMP1..4 ==	TIMP1..4 all ↑ [38]
	MMP2 == MMP15 ↑, MMP24 ↑	MMP2 ↑ [38] MMP15 ? MMP24 ?
	ADAM12 ↑ ADAM19 ↑	ADAM12 ↑ [47]
	SERPINE1 ↑, SERPINE2 ↑	SERPINE1 ↑ [39], SERPINE2 ?
	SPARC ↑	SPARC ?
	TSPAN2 ↑, TSPAN13 ↑, TSPAN15 ↑	TSPAN2 ? TSPAN13 ? TSPAN15 ?
	LOXL1 ↑ LOXL2 ↑	LOXL1 ↑
ECM components	COL1A1 ↑ COL4A1 ↑ COL4A2 ↑ COL4A4 ↑ COL5A2 ↑ COL14A1 ↓	COL4A1 ↓ COL4A2 ↓ [4] (lens tissue)
homocysteine metabolism	not enriched	-
mitochondrial dysfunction	not enriched	-
impaired autophagy	not enriched	-

## Data Availability

Data is contained within the article. The raw RNA-Seq data was deposited and released in the SRA database (Study: https://www.ncbi.nlm.nih.gov/bioproject/882267 (created on 26 September 2022)).

## Supplementary Materials

The following supporting information can be downloaded at: https://www.mdpi.com/article/10.3390/biom12111693/s1, Figure S1: LOXL1 SNPs and sex of donors in this study; Figure S2: Genes associated with XFS from genome-wide association studies (GWAS); Table S1: RT-qPCR primer sequences; Table S2: Genelist resulting from RNA-Seq, sorted by adjusted *p*-value; Table S3: Enrichment results.
