# Supplementary material for: Genome-Wide RNA Sequencing of Human Trabecular Meshwork Cells Treated with TGF-β1: Relevance to Pseudoexfoliation Glaucoma
Source: Biomolecules. 2022 Nov 15;12(11):1693. doi: 10.3390/biom12111693 (PMC9687758; doi:10.3390/biom12111693)
Supplement: Supplementary file 1 [file biomolecules-12-01693-s001.zip › Figure_S1.pdf]

biomolecules

MDPI

Supplemental Figure S1 of Genome-wide RNA sequencing of human trabecular meshwork cells treated with TGF-β1 : relevance to pseudoexfoliation glaucoma

A

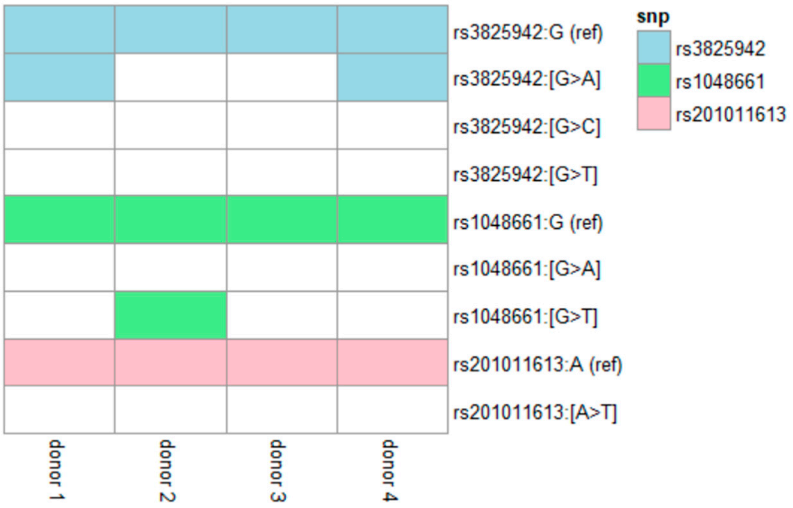

B

donor	sex
1	M
2	F
3	F
4	M

Figure S1. LOXL1 SNPs and sex of donors in this study (A) LOXL1 genotyping for several SNPs. Donors 1 and 4 were heterozygous for rs3825942:[G>A] while donor 2 was heterozygous for SNP rs1048661:[G>T]. Donor 3 was wild-type and did not have any of the studied SNPs. For rs201011613 all donors were wild-type (B) donor sex.
