# Supplementary material for: Genome-Wide RNA Sequencing of Human Trabecular Meshwork Cells Treated with TGF-β1: Relevance to Pseudoexfoliation Glaucoma
Source: Biomolecules. 2022 Nov 15;12(11):1693. doi: 10.3390/biom12111693 (PMC9687758; doi:10.3390/biom12111693)
Supplement: Supplementary file 1 [file biomolecules-12-01693-s001.zip › Figure_S2.pdf]

biomolecules

Supplemental Figure S2 of Genome-wide RNA sequencing of human trabecular meshwork cells treated with TGF-β1 : relevance to pseudoexfoliation glaucoma

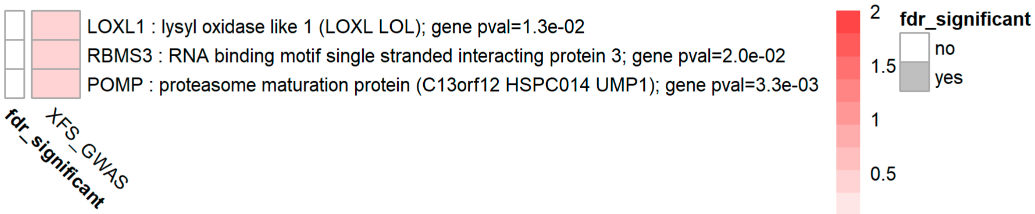

Figure S2. Genes associated with XFS from genome-wide association studies (GWAS). Heatmap showing genes with nominal p-value $p<0.05$ for genes associated with genome-wide association studies for pseudoexfoliation syndrome (XFS). Heatmap colours correspond to fold change expressed as log2FC which has been limited to a range of [0,2] for clarity. The annotation bar on the left shows if the genome-wide false discovery rate has an FDR<0.05 (in which FDR is the BH corrected p-value) for a particular gene which is the case for none of the genes shown.
