# Supplementary material for: Genome-Wide RNA Sequencing of Human Trabecular Meshwork Cells Treated with TGF-β1: Relevance to Pseudoexfoliation Glaucoma
Source: Biomolecules. 2022 Nov 15;12(11):1693. doi: 10.3390/biom12111693 (PMC9687758; doi:10.3390/biom12111693)
Supplement: Supplementary file 1 [file biomolecules-12-01693-s001.zip › Table_S1.pdf]

biomolecules MDPI

Genome-wide RNA sequencing of human trabecular meshwork cells treated with TGF-β1 : relevance to pseudoexfoliation glaucoma : supplemental Table S1

Table S1. RT-qPCR primer sequences

gene symbol	sequences
TSPAN2	**F** 5′-CCAGAGTATTTCTATGTGGGGCTG-3′<br>**R** 5′-AGCAAATATCACCAGGAGGCAG-3′
THBS1	**F** 5′-GACTCCGCATCGCAAAGGG-3′<br>**R** 5′-GAGGACACTGGTAGAGCTGGAG-3′
ADAM12	**F** 5′-ATCAGTGTCTTCGGCGTTCA-3′<br>**R** 5′-GGCAATTCTTCCTGTTGTTACATACC-3′
IER3	**F** 5′-CTCGAGTGGTCCGGCG-3′<br>**R** 5′-ACGATGGTGAGCAGCAGAAA-3′
LOXL1	**F** 5′-ACAGCACCTGTGACTTCGGCAA-3′<br>**R** 5′-CGGTTATGTCGATCCACTGGCA-3′
ADAM19	**F** 5′-CGGGCCCACCTCGAA-3′<br>**R** 5′-CCGTTTCATTCTGCGAGGTT-3′
NOX4	**F** 5′-CACAGACTTGGCTTTGGATTTC-3′<br>**R** 5′-GGATGACTTATGACCGAAATGATG-3′
CCN2	**F** 5′-GCTTACCGACTGGAAGACACG-3′<br>**R** 5′-CGGATGCACTTTTTGCCCTT-3′
GAPDH	**F** 5′-GGAGCGAGATCCCTCCAAAAT-3<br>**R** 5′-GGCTGTTGTCATACTTCTCATGG-3′
